# Health Tract, No. 59

**Published:** 1862-02

**Authors:** 


### HEALTH TRACT, No. 59.

APPLES.—The apple is perhaps more useful than all the other fruits in nature. Beyond them all, it is durable, prolific, easy of culture, and capable of such a variety, in its mode of preparation for the table, that a small volume might be written about it. The time required to digest a piece of roasted pork is five hours and a half; about equal to a piece of boiled tendon, (white leather,) which is almost leathery, or a lump of boiled beef-suet; while a sweet, mellow, raw apple is digested, passed out of the stomach, and enters the circulation to nourish and strengthen, in an hour and a half, being exceeded in easiness of digestion only by boiled rice, pigs' feet or tripe soused, and whipped eggs, all of which are digested in one hour. Sweet apples are not valued as they ought to be, because they do not "cook well;" but to be eaten raw, there is scarcely any thing more "delicate," that is, so easily received into the system, requiring so little stomach power in appropriating it to the nourishment of the body. One good method of cooking apples, is to peel them and take out the core, without dividing the fruit; put them in a dish, pour over them a few table-spoonfuls of water; bake until delicately brown, and eat with cream and sugar, as a dessert, for dinner. This is incomparably preferable to the sodden dumpling or the greasy pie. Mrs. F. D. Gage, one of the most notable housewives in the nation, says: "Pare the apples and quarter them, placing them in a tin plate with the core side up; if dried apples, a little water is added; they are then set in the oven, which is always hot at meal-time, and roasted; when done, they are slid on a common plate, and sprinkled with sugar; to be eaten warm, with bread and butter and cakes. It would require canned fruit of extra flavor to tempt me from the apple-dish, if thus prepared. Strawberries or half-ripe peaches are not to be talked of the same day."

For lunches at school or at home, for convenience and cleanliness, to put in the pocket while traveling, or on an excursion, or when expecting to be absent from home over a meal, the apple is without an equal; while as a dessert it might well supersede all the cakes, pies, jellies, dumplings, and "tarts" ever invented. If a tithe of the money expended in easily dispensable articles of apparel, or mere personal gratifications in the shape of snuff, cigars, chewing-tobacco, home-made wines and cordials, or of useless trinkets of jewelry, or unsubstantial, unremunerative amusements, was devoted to the purchase of a bountiful supply of apples in the fall, for family use, without stint, there would be found a most welcome increment in family health in the spring, and a diminution of doctors' bills, especially gratifying to all prudent and calculating "pater-familias." To every householder we say, wear an old coat another year, do with one silk dress less, skimp yourself in pork, ham, bacon, and even roast beef, rather than fail to put half a dozen barrels of prime apples in your cellar this fall.

